# Waterborne protozoan and microsporidian parasites in Eurasian beavers (*Castor fiber*)

**DOI:** 10.1016/j.ijppaw.2025.101050

**Published:** 2025-02-27

**Authors:** Júlia Šmigová, Viliam Šnábel, Serena Cavallero, Ľubomír Šmiga, Ingrid Papajová, Bohumil Sak, Nikola Holubová, Martin Kváč

**Affiliations:** a Institute of Parasitology, Slovak Academy of Sciences, Hlinkova 3, 040 01, Košice, Slovak Republic; b University of Veterinary Medicine and Pharmacy in Košice, Komenského 73, 041 81, Košice, Slovak Republic; c Department of Public Health and Infectious Diseases, Sapienza University of Rome, Piazzale Aldo Moro 5, 00185, Rome, Italy; d Institute of Parasitology, Biology Centre of the Czech Academy of Sciences, České Budějovice, České Budějovice, Czech Republic; e Faculty of Agriculture and Technology, University of South Bohemia, České Budějovice, Czech Republic

**Keywords:** Protozoan, Zoonoses, Gastrointestinal parasites, Molecular analysis, Protected animals, Rodents

## Abstract

The Eurasian beaver (*Castor fiber*) is an expanding species in Europe in recent decades due to reintroductions and natural population growth. Beavers expanded rapidly in the second half of the 20th century, and their expansion was particularly rapid in the Danube basin. Nowadays, the majority of the continuous population located in the central and eastern parts of the continent and a large disjunct population in Norway and Sweden. Despite the increasing population size, the role of the beaver as a source of waterborne pathogens is not firmly established or is often inferred from circumstantial data. In order to extend knowledge about the composition of the parasite fauna of beavers occurring in Slovakia, 21 faecal samples taken near their burrows from three sites (located in the Topľa, Poprad and Danube river basin) were examined microscopically and by polymerase chain reaction (PCR). PCR-positive specimens were further examined by DNA sequencing. Parasites were detected in 21% of the examined beavers, specifically the protozoa *Cryptosporidium* spp. (n = 2), *Blastocystis* sp. (n = 1), and microsporidia *Enterocytozoon bieneusi* (n = 1) and *Encephalitozoon* spp. (n = 1). Using the sequence analysis, two variants of *Cryptosporidium proliferans*, a new subtype of *Blastocystis* sp., genotype D of *E. bieneusi* and *Encephalitozoon intestinalis* were identified. A putatively novel *Blastocystis* subtype (ST), originated from a site near the Danube river (southwestern Slovakia), was proposed based on high genetic divergence from the closest described subtype ST12 (11.9%) and unique phylogenetic position in a clade composed of ST's 35–38. The increased risk of zoonotic transmission or transmission to other animals was particularly evident in the site near the Topľa river (northeastern Slovakia), where fungal spores of zoonotic genotype D of *E. bieneusi* and *E. intestinalis*, together with oocysts of the potentially zoonotic *C. proliferans*, were found.

## Highlights

• Beavers as a source of potential zoonotic pathogens.
• Finding of the species of the gastric group *Cryptosporidium proliferans* in beavers.
• First *Blastocystis* sequence obtained from a beaver worldwide.
• Description of *E. intestinalis* and *E. bieneusi* (genotype D) in Eurasian beavers.

## Graphical abstract

**Figure:**
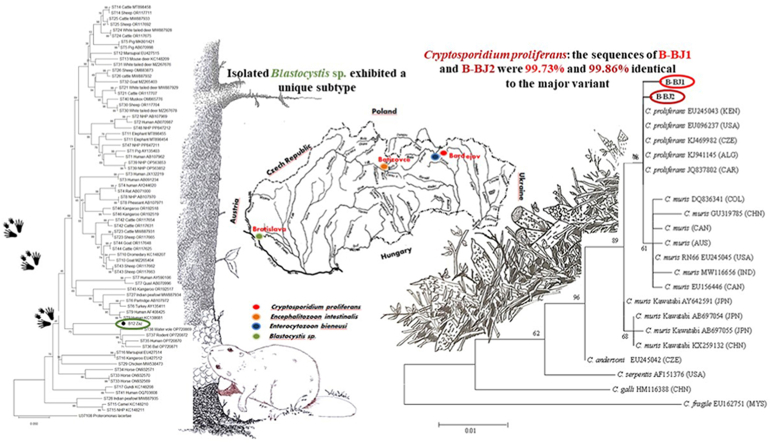

## 1 Introduction

A number of species of protozoan parasites play an important role in the development of serious diseases that have significant implications in human and veterinary medicine. Wild animals, especially rodents, are considered potential reservoirs of parasitic protist in surface waters such as *Cryptosporidium*, *Blastocystis* sp., *Giardia duodenalis* and microsporidia from the genera of *Enterocytozoon* and *Encephalitozoon* (Robertson et al., 2019). These protists are regarded to be transmitted via the fecal-oral route, which involves direct contact with infected hosts and ingestion of contaminated food or water (Fayer et al., 2006).

The main clinical signs of cryptosporidiosis, giardiasis and blastocystosis include diarrhoea and other abdominal disorders such as abdominal pain, flatulence, nausea, bloating and vomiting (Osman et al., 2016). While *Giardia* and *Cryptosporidium* can directly induce disease by damaging and inflaming the gut epithelium, *Blastocystis* affects the gut microbiota of the host by changing its wider species composition, thereby indirectly influencing host health and disease state (Savioli et al., 2006; Burgess et al., 2017).

*Cryptosporidium* was reported to be responsible for more than 8 million cases of foodborne illness worldwide annually and causes up to 20% of all cases of diarrhoea in children in developing countries (Mosier and Oberst, 2000; Ryan et al., 2018). Currently, at least 49 *Cryptosporidium* species and approximately 120 genotypes are recognized in this diverse group, with the greatest diversity reported in rodents, where 14 species have been described (Ryan et al., 2021; Ježková et al., 2023). So far, 22 *Cryptosporidium* species and genotypes have been identified in humans, with *Cryptosporidium hominis* and *Cryptosporidium parvum* responsible for more than 90% of human infections (Helmy and Hafez, 2022).

*Blastocystis* species (sp.) is probably the most widespread human intestinal parasite, with an estimated more than one billion infections worldwide (Andersen and Stensvold, 2016). This eukaryotic protist belongs to the stramenopile clade of heterokonts and can infect a variety of mammalian and avian species (Stensvold and Clark, 2016; Boutahar et al., 2023). The pathogenicity and public health significance of *Blastocystis* is still unclear and controversial, as the parasite is highly represented in asymptomatic healthy individuals but can shift from being mutualistic to becoming pathogenic when the host immune system is immunocompromised (Salehi et al., 2021). Almost 90% of human isolates belong to the genetically defined subtypes ST1-ST4, with the majority being attributable to ST3 (Maloney et al., 2023).

Accumulated phylogenetic evidence suggested that obligate intracellular Microsporidia parasites are actually highly reduced fungi grouped with *Cryptomycota* in the basal branch formed by zoosporic fungi, which represents a sister group to the fungal kingdom (James et al., 2013; Spatafora et al., 2017). They are emerging opportunistic pathogens that can infect many invertebrates and vertebrates, including humans, domestic and wild animals (Didier and Weiss, 2006). Microsporidial infections range from asymptomatic to even fatal in immunodeficient people, with a wide variety of clinical symptoms and diarrhoea as the main clinical symptom (Sak and Kváč, 2021).

*Enterocytozoon bieneusi* and three *Encephalitozoon* species, *Encephalitozoon cuniculi*, *Encephalitozoon hellem* and *Encephalitozoon intestinalis*, are the four most common microsporidian species capable of human infection (Santín and Fayer, 2011). Among them, *E. bieneusi* is responsible for most of the human clinical manifestations (Wang et al., 2024). More than 500 genotypes of *E. bieneusi* were described in mammals and birds, divided into 15 phylogenetic groups, with Groups 1 and 2 being the largest and confined to human infections in multiple geographic areas (Li and Xiao, 2021; Nourrisson et al., 2024). On the other hand, *E. bieneusi* genotypes belonging to Groups 3 to 11 mostly have a restricted host range, indicating their limited zoonotic potential and a minor or unknown public health treat (Li et al., 2019). In the *Encephalitozoon* microsporidians, mammals are frequently infected with *E. cuniculi* (prevalent mainly in the rabbit population), *E. intestinalis* (primarily recorded in farm animals, companion animals and wildlife), and less often with *E. hellem*, for which birds are the primary reservoir (Wilczyńska et al., 2022).

After the extinction of the Eurasian beaver (*Castor fiber*) in Slovakia in the 19th century, thanks to reintroduction and translocation projects in neighboring countries, it returned to the country in 1977 from Austria along the Danube river to the southwestern part of the country, and in 1981 from Poland to the Ondava basin in eastern Slovakia (Valachovič, 2012). Suitable conditions for the beaver expansion across the country are currently on 56% of the Slovak territory, where it inhabits stagnant and flowing waters of all categories (Drengubiak, 2019; Jarčuška et al., 2020). In Slovakia, the beaver population is continuously increasing; in 2023, 700 beaver individuals were counted only in hunting grounds (NFCSR, 2023). Beavers are regarded as ‘ecosystem engineers’, capable of changing the dynamics of the water flow and its purification, moderating extreme events as drought or floods, and increasing the biodiversity of wetland habitats (Thompson et al., 2020). However, they are also important sources of parasitic protozoa in surface waters and raise public health concerns especially when streams and ponds are in close proximity to municipal drinking water supplies or recreational water sites (Fayer et al., 2006; Girling et al., 2019).

While microsporidia and *Blastocystis* have not yet been studied in Eurasian beavers, both *Cryptosporidium* and *Giardia* have already been recorded in Europe, but the number of studies is limited (e.g., Paziewska et al., 2007; Turillazzi et al., 2024). The risk of reintroducing historically native host species back into original areas is often neglected and the Eurasian beaver with its often understudied parasite fauna may pose such a hazard (Benovics et al., 2022). The aim of the present study was therefore to analyse the species distribution and prevalence of the parasitic protozoa in the European beaver population of Slovakia originating from three distinct areas using molecular and microscopic methods.

## 2 Material and methods

### 2.1 Source of specimens

A total of 21 faecal samples were collected at nearby sites close to beaver burrows, specifically 16 samples were taken from the Topľa river near the village Rokytov in the Bardejov district (northeastern Slovakia) and 4 samples in the Velický brook (tributary of the Poprad river) near the village Batizovce, Poprad district (north Slovakia). In addition, one sample originating from beaver individual was taken from the rescue station Zázrivá, where the animal was placed after an attack by older beaver individuals near the Danube river in the Bratislava district (southwestern Slovakia). All faecal samples were transferred to the laboratory.

### 2.2 Microscopy analyses

For coprological examination, the centrifugal flotation method was employed using a sucrose flotation solution with a specific gravity of 1.27. Each faecal sample obtained from an individual animal was also examined for the presence of *Cryptosporidium* oocysts using the aniline-carbol-methyl violet (ACMV) staining method (Miláček and Vítovec, 1985) followed by microscopic examination at 1000× magnification.

### 2.3 Molecular analyses

Genomic DNA (gDNA) was extracted from 200 mg of stool by bead disruption for 60 s at a speed of 5.5 m/s using 0.5 mm glass beads in a Qiagen TissueLyser (Qiagen, Hilden, Germany). The gDNA was then isolated/purified using ExgeneTM Stool DNA mini kit (GeneAll Biotechnology Co. Ltd., Seoul, Korea) according to the manufacturer's instructions.

For the detection and typing of *Cryptosporidium* spp. and *Blastocystis* sp., PCR protocols adapted from Xiao et al. (1999) and Scicluna et al. (2006) were used to amplify partial sequences of the gene encoding the small subunit rRNA (SSU). For *Cryptosporidium* spp., primers used for primary and secondary amplification (referred to as F1/R1, F2/R2 primers) were those described by Xiao et al. (1999). The scorable length of the PCR fragment in the two examined *Cryptosporidium* samples was 730 bp. For *Blastocystis* sp., amplification was employed with RD3/RD5 primers described by Clark (1997) for primary PCR and with Bla1/Bla2 primers described by Liu et al. (2022) for secondary PCR. The scorable length of the PCR fragment in the examined *Blastocystis* sample was 658 bp.

For the genetic characterization of *Encephalitozoon* spp. and *E. bieneusi*, the internal transcribed spacer (ITS) was amplified according to the PCR protocol of Katzwinkel-Wladarsch et al. (1996). In this protocol, MSP-I/MSP-3 primers for primary PCR and MSP-2B/MSP-4B primers for secondary PCR in case of *E. bieneusi*, and MSP-I/MSP-3 primers for primary PCR and MSP-2A/MSP-4A primers for secondary PCR in case of *Encephalitozoon* spp., being used in the present study, were described. The scorable length of the examined sample belonging to *Encephalitozoon* spp. was 231 bp, whereas the sample belonging to *E. bieneusi* produced a fragment with the size of 381 bp.

The primary PCR mixtures were composed of 2 μl gDNA, 2.5 U Taq DNA polymerase (Dream Taq Green DNA Polymerase, Thermofisher Scientific, Waltham, MA, USA), 1 × PCR buffer, 6 mM MgCl2 (for SSU of *Cryptosporidium* spp.), or 1 mM MgCl2 (for SSU of *Blastocystis* sp.), or 3 mM MgCl_2_ (for ITS of *Encephalitozoon* spp. and *E. bieneusi*), 200 μM each deoxynucleoside triphosphate, 100 mM each primer, and 2 μl of non-acetylated bovine serum albumin (BSA; 10 mg/ml; New England Biolabs, Beverly, MA, USA) in a 30 μl reaction volume.

Each PCR consisted of 35 cycles of denaturation at 94 °C for 45 s, annealing for 45 s at 50/55 °C for SSU of *Cryptosporidium* spp., 54/54 °C for SSU of *Blastocystis* sp., 55/55 °C for ITS of *Encephalitozoon* spp., 55/57 °C for ITS of *E. bieneusi* and extension at 72 °C for 60 s; an initial denaturation step consisting of incubation at 94 °C for 5 min and a final extension step consisting of incubation at 72 °C for 10 min were also included.

Negative controls consisted of molecular-grade water, and positive controls included DNA from *C. serpentis*, *E. hellem*, *E. bieneusi* genotype PtEbIX and *Blastocystis* sp. subtype ST3. The secondary PCR products were visualized in a 1.5% agarose gel stained with ethidium bromide. Subsequently, the PCR products were excised from the gel, purified using the Gen Elute Gel Extraction Kit (Sigma, St. Louis, MO, USA), and subjected to bidirectional sequencing.

DNA sequencing was performed on an automated DNA sequencer ABI prism 3700 at the University of Veterinary Medicine and Pharmacy, Košice, Slovakia. Obtained DNA sequences were compared with reference sequences in the GenBank database using the BLASTn nucleotide program. Nucleotide alignments were constructed on the webPRANK server (Löytynoja and Goldman, 2010), which correctly handles insertions and avoids overestimation of deletion events. For *Cryptosporidium* and *Blastocystis* samples, it was needed to carry out phylogenetic analyses to generate high-resolution trees that clarified relationship of our isolates to close organisms. As a result, a maximum likelihood (ML) tree for *Cryptosporidium* samples and a neighbor-joining (NJ) tree for *Blastocystis* samples were selected by the hierarchical likelihood ratio test with 1000 bootstrap pseudoreplicates, using the MEGA7 software (Kumar et al., 2016).

## 3 Results

**Fig. 1:**
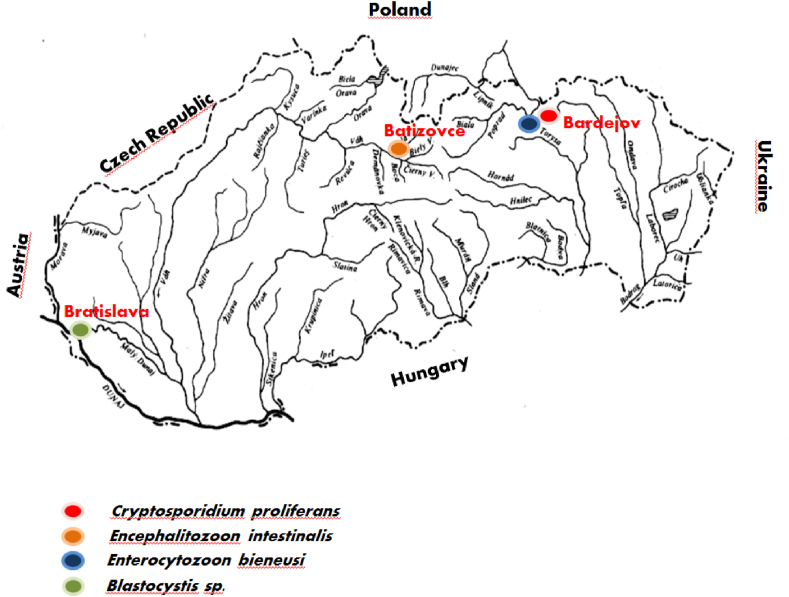
Map of positive samples on different localities.

Twenty-one samples of beaver faeces were collected from three geographic locations in Slovakia (Fig. 1), and subsequently analysed microscopically and genetically. Two protozoan species of gastrointestinal parasites were detected microscopically. We found *Cryptosporidium* oocysts (in 2 samples) and the cysts of *Blastocystis* (in 1 sample). Microsporidia spores and *Giardia* cysts were not found microscopically. Twenty-one stool samples were then subjected to molecular analyses, which showed the presence of specific DNA of *Cryptosporidium* spp. (n = 2), *Blastocystis* sp. (n = 1), *Encephalitozoon* spp. (n = 1) and *E. bieneusi* (n = 1) by detecting PCR products. Further DNA sequencing revealed the identity of protozoan parasites in beavers at the species/genotype/subtype levels.

**Fig. 2:**
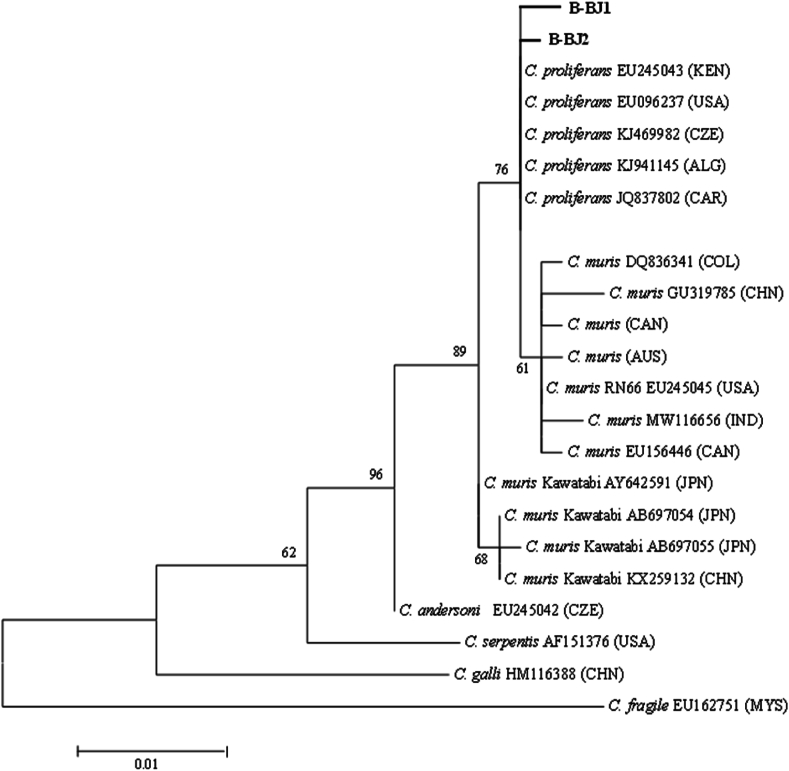
Phylogram generated from sequences of the partial small subunit of ribosomal RNA (734 bp) by the maximum likelihood (ML) method based on the Tamura-Nei model in MEGA7, showing relationships between the examined beaver *Cryptosporidium* isolates from Slovakia (B-BJ1, B-BJ2) and GenBank-retrieved related sequences. The amplified region of the Slovak *Cryptosporidium* samples had the length 730 bp. The nucleotide substitution model with the lowest BIC score T92+G (G = 0.05) was selected to run ML inference. The scale bar refers to a phylogenetic distance of 0.01 nucleotide substitutions per site. Numbers next to the branches indicate bootstrap values. Countries of origin indicated using abbreviations: KEN - Kenya; USA - United States of America; CZE - Czech Republic; ALG - Algeria; CAR - Central African Republic; COL - Colombia; CHN - China; CAN - Canada; AUS - Australia; IND - India; JPN - Japan; MYS – Malaysia.

For *Cryptosporidium* spp. typing, a phylogenetic maximum likelihood tree inferred from SSU sequences showed that the beaver isolates B-BJ1 and B-BJ2 (both originating from the Topľa river in northeastern Slovakia) clustered with the *Cryptosporidium proliferans*, being located in the branch supported with a bootstrap value of 76%. The B-BJ1 sample differed in two nucleotide substitutions (75G/A, 481 A/G) from a structure of *C. proliferans* detected so far (having GenBank records for isolates from the Czech Republic (GenBank accession number KJ469982), Kenya (EU245043, KR090615), Central African Republic (JQ837802), Algeria (KJ941145), USA (EU096237), whereas one substitution (255 A/G) was detected in the B-BJ2 isolate compared to the *C. proliferans* profile. Closely related *Cryptosporidium muris* isolates were grouped in an adjacent major cluster (bootstrap support of 64%, Fig. 2), consisting of the most common *C. muris* genotype (represented by the RN66 isolate from brown rat, USA (EU245045) and representatives of the remaining variable profiles with 1–3 single nucleotide polymorphisms (SNP's), identified worldwide. The second *C. muris* variant, associated with the ‘Kawatabi strain’ recorded so far only in East Asia (Japan (AY642591, AB697054, AB697055) and China (KX259132), which might have arisen due to geographic separation, formed an adjoining cluster receiving a bootstrap support of 65%. The sequences of B-BJ1 and B-BJ2 were 99.73% and 99.86% identical to the major variant of *C. proliferans*. In relation to the common *C. muris* type, the matched identities were 99.6% and 99.7%. The beaver isolates shared average 99.3% and 99.4% identities with the group of the putative second variant of *C. muris* (Kawatabi strain). In addition, 98.8% and 98.9% of the nucleotides of the two examined isolates were identical *to Cryptosporidium andersoni*.

**Fig. 3:**
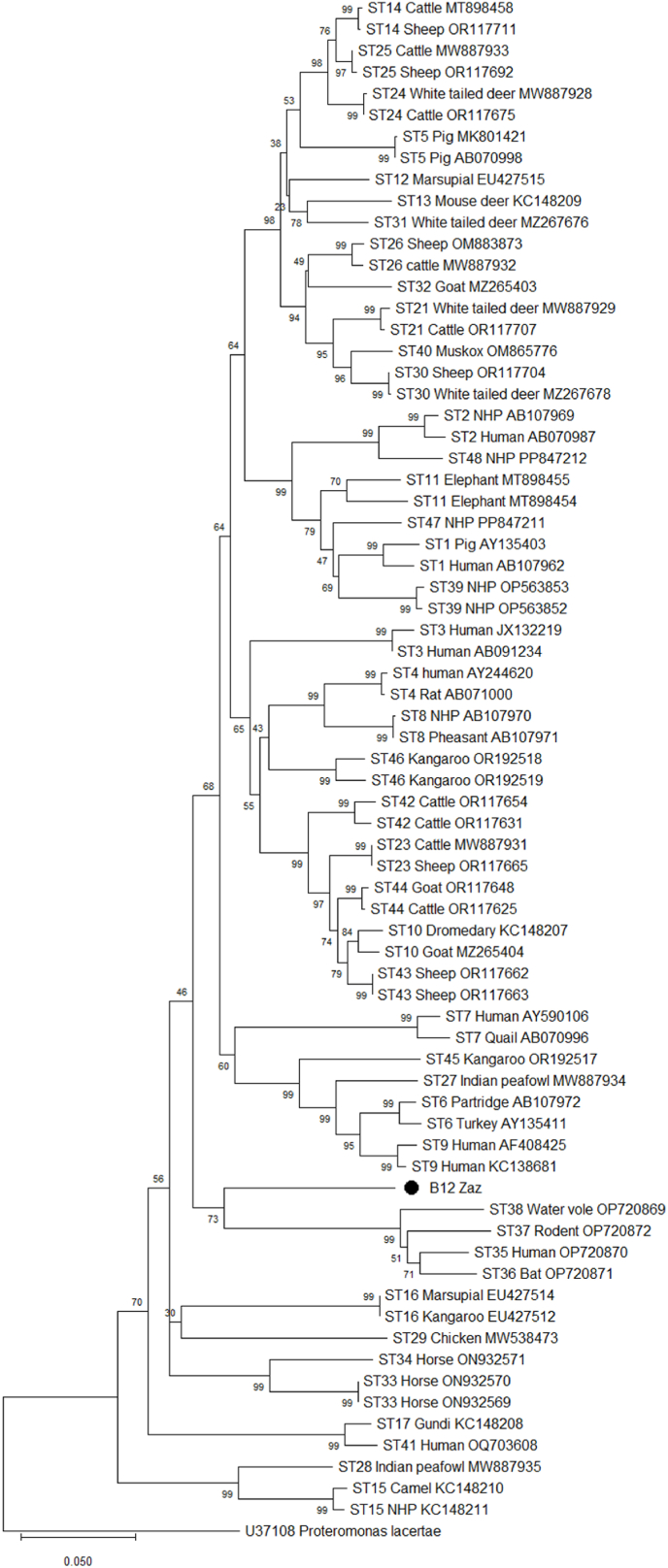
Phylogram generated from sequences of the partial small subunit of ribosomal RNA (739 bp) by the neighbor-joining (NJ) method, showing relationships between the examined beaver *Blastocystis* isolate from Slovakia (B12-Zaz) and GenBank-retrieved *Blastocystis* subtypes (the two most common sequences for each subtype if available). The amplified region of the Slovak *Blastocystis* sample had the length 658 bp. The nucleotide substitution model with the lowest BIC score TN93 and G:0.45 was selected to run NJ inference. The scale bar refers to a phylogenetic distance of 0.05 nucleotide substitutions per site. Numbers next to the branches indicate bootstrap values. ‘NHP’ host means ‘non-human primate'.

In the framework of *Blastocystis* sp. subtype classification, a phylogenetic neighbor-joining tree of the barcoding region (658 bp) of SSU showed that cysts isolated from the beaver isolate (designated as B12-Zaz and originating from the Danube river, southwestern Slovakia) exhibited a unique *Blastocystis* subtype (ST) clustering with ST's 35–38, which were described in humans from Brazil, wild mammals from Mexico, and captive water voles from the UK (Fig. 3). The resulting clade, in which B12-Zaz and the three previously described isolates (ST's 35–38) were placed in different sub-branches, was supported by a relatively high bootstrap value of 73. The BLASTn search showed that the beaver sample shared the highest genetic identity (88.1%) with the unpublished ST12 sequence of *Blastocystis* sp. from the northern swamp wallaby, followed by samples assigned to ST5 (identity ranging from 87.4 to 87.7%), which are, however, very different from the sequences of our sample.

At the ITS locus, *E. bieneusi* and *Encephalitozoon* isolates (designated as B10, B20; samples came from the Bardejov district, northeastern Slovakia and from the Velický brook (tributary of the Poprad river) near the village Batizovce (north Slovakia) shared 100% identity with genotype D of *E. bieneusi* (available under the GenBank accession number AF101200) and *Encephalitozoon intestinalis* (MG570080), respectively (data not shown graphically).

The sequences of *Cryptosporidium* samples (denoted as B-BJ1, B-BJ2) were submitted to GenBank under accession numbers OM236545 and OM236667, *Blastocystis* sp. sample (denoted as B12-Zaz) under accession number PP094780, *E. bieneusi* sample (denoted as B10) under accession number PP782303, and *E. intestinalis* sample (denoted as B20) and under accession number PP782304.

## 4 Discussion

The aquatic environment plays a major role in the distribution and spread of pathogens, especially protozoa of the genera *Cryptosporidium*, *Giardia*, *Blastocystis*, and microsporidia of the genera *Enterocytozoon* and *Encephalitozoon*. Their transmissive stages (oocysts, cysts, spores) are environmentally robust and ubiquitous in aquatic habitats, posing a health threat to humans and animals worldwide, mainly in areas with inadequate or contaminated water sources (Graczyk et al., 2008). In addition to secondary surface contamination by water erosion, agricultural activities, inconsistencies in water distribution systems and faecal contamination (particularly from humans and livestock), surface waters can also be directly contaminated by aquatic and semi-aquatic animals (Ma et al., 2022). Information regarding the parasitological status of beavers as semi-aquatic rodents widespread in most of the northern hemisphere is currently rather limited.

In the global rodent population, which represents more than 40% of species diversity within the class Mammalia, nearly 10% of species are reservoirs of zoonotic pathogens (Han et al., 2015). The overall worldwide prevalence of *Cryptosporidium* spp. in wild rodents was estimated by Zhang et al. (2022) as 19.8%. In this survey, in the population of Eurasian beaver in Slovakia (a semi-aquatic rodent belonging to the family Castoridae), *Cryptosporidium* oocysts occurred in 9.5% (2/21) of stool samples. A lower infection rate (4.8%, 1/21) in beavers was also found here in the case of *Blastocystis* sp., whereas for a global rodent population Barati et al. (2022) estimated a prevalence of 18%. The minor rates of infection with protozoan parasites observed in Slovak beavers compared to some established beaver populations worldwide, e.g. those in northwest Poland and the Pacific Northwest, USA (Sroka et al., 2015; Li et al., 2020). This can be partly attributed to the fact that, in connection with the successful reintroduction of beavers in Slovakia, the increase in beaver abundance and their expansion into new suitable habitats has only recently begun to manifest itself (Lazár et al., 2023). Nevertheless, the number of beavers recorded as infected with protozoa or microsporidia was relatively high in the present study (23.8%, 5/21). In a previous study on Eurasian beavers in Slovakia with sampling carried out in 2018–2019, Bystrianska et al. (2021) recorded a 10.5% prevalence of *Cryptosporidium* spp. in faecal samples. *Cryptosporidium* spp. oocysts in beavers were reported in Poland (neighboring to Slovakia), where a prevalence of 10.5% (2/19) was found by Siński et al. (1998) in faecal samples taken in the Kampinos National Park and the Research Station in Popielno (central and northeastern Poland), while in a close area Suwałki district to Popielno a prevalence of 19.2% (10/52) was recorded by Paziewska et al. (2007). In another study conducted in northeastern Poland in 2010–2014 in the Masurian Lake District by Sroka et al. (2015), a prevalence of up to 45.6% (36/79) was measured in water samples collected in 14 locations situated in a vicinity of beaver lodges that pose a risk to human health. Elsewhere in Europe, following the translocation and release of 16 wild beavers from Norway to Scotland, one kit already born in Scotland was tested as positive for *Cryptosporidium* oocysts in a faecal sample obtained post-mortem (Goodman et al., 2017). Genetically, in a related North American beaver (*Castor canadensis*), *Cryptosporidium ubiquitum* was identified in three isolates from USA (Feng et al., 2007; Tang et al., 2016), and *Cryptosporidium* sp. beaver genotype in two isolates from USA (Feng et al., 2007). Both these variants belong to the larger intestinal group of the genus *Cryptosporidium.* To our knowledge, our records of *C. proliferans* in two beavers from Slovakia are thus the first findings among beaver-derived isolates associated with the gastric *Cryptosporidium* group.

Both beaver isolates from northeastern Slovakia here analysed exhibited a nucleotide polymorphism in the partial SSU gene associated with one/two specific nucleotides. Unlike this, *C. proliferans* isolates from three continents (Europe, Africa, North America) and five naturally infected hosts (East African mole rat, Eastern gray squirrel, domestic horse, donkey, African buffalo) belonging to Rodentia, Arctiodactyla and Perissodactyla orders, before sequenced in SSU, showed homogeneous genetic profiles (Feng et al., 2007; Sak et al., 2013; Laatamna et al., 2015; Kváč et al., 2016). The similar variability in SSU gene has been reported in other *Cryptosporidium* spp. such as *C. parvum*, *C. ditrichi*, *C. andersoni*, *C. microti* (Le Blancq et al., 1997; Čondlová et al., 2019). Although the results of this and previous studies indicate a broad host specificity of *C. proliferans*, this species has been detected in only a limited number of studies and little is known about its epidemiology (Kváč et al., 2016; Valigurová et al., 2018). While the related gastric species *C. muris* and *C. andersoni* have documented zoonotic potential, there is no direct evidence to date that *C. proliferans* is infectious to humans (Gatei et al., 2002; Leoni et al., 2006; Zahedi et al., 2016). However, Li and Atwill (2021) concede that variants with ≥99.5% sequence homogeneity to a known zoonotic *Cryptosporidium* species/genotype in a reasonably long SSU fragment can be considered as potentially infectious to humans, which is consistent with the relatedness of our *C. proliferans* variants to the common *C. muris* type (the identities to isolate RN66 were 99.6% and 99.7%).

Because of the great deal of genetic diversity contained in the *Blastocystis* sp. complex with multiple genetic groupings called subtypes, surveys of the diversity of these intestinal protist parasites from a variety of hosts and geographic locations are of interest to better recognize their epidemiology and zoonotic potential. A subtyping system based on sequencing of the SSU gene is currently used to distinguish between genetic variants of *Blastocystis* sp. (Stensvold et al., 2007; Stensvold and Clark, 2020). Using this scheme, at least 44 subtypes (ST1–ST17, ST21, and ST23-ST44) have been so far established (Maloney and Santin, 2021; Santín et al., 2024). No strict associations between the subtypes and hosts have been reported, although moderate host specificity was observed (Barati et al., 2022). In the present study, a candidate for a novel subtype originating from a faecal sample taken from a watershed of the Danube river (southwestern Slovakia) was indicated based on an 11.9% difference to the sequence of the closest known subtype (ST12). This meets the condition of at least 4% divergence demonstrated across the potentially novel sequence according to the current guidelines for *Blastocystis* subtyping classification (Stensvold and Clark, 2020). To our knowledge, this is the first *Blastocystis* sequence obtained from a beaver host worldwide. In previous studies conducted on *Blastocystis* molecular typing in Slovakia, in a group of hemodialysis patients, *Blastocystis* sp. was identified in 13 patients (24.5%), with a predominant prevalence of the ST3 subtype (n = 9), followed by ST1 (n = 3) and ST2 (n = 1) (Hatalová et al., 2024). In brown bears surveyed in the Poloniny National Park (east of Slovakia), 50 % (8/16) of the faecal samples were positive and exhibited ST3 subtypes (Valenčáková et al., 2014). In the ZOO Košice (eastern Slovakia), *Blastocystis* sp. was found in 20% (5/25) of housed animals, specifically ST5 (n = 3) in two birds (Bernier's teal, marabou stork) and in a black-and-white ruffed lemur, ST7 in a ring-tailed lemur, and ST12 in a wild boar. In two farms in eastern Slovakia, the ST5 subtype (globally predominant in pigs and wild boars) was identified in all 12 positive pigs, accounting for a prevalence of 12% (12/100) (Danišová et al., 2022, Danišová and Valenčáková, 2021). All these subtypes (ST1-ST3, ST5, ST12) recorded in Slovakia were previously also found in humans in a global context (Rudzińska and Sikorska, 2023).

A number of genotypes have been identified within the microsporidian species of *E. bieneusi* and *E. intestinalis*, many of which exhibit broad host specificity and have been described in humans, livestock and wild vertebrates (Robertson et al., 2019; Sak and Kváč, 2021). In our study, one of 21 animals (4.8%) was positive for *E. bieneusi*, detected in the Topľa river and categorized as carrying the genotype D (designated as such by Rinder et al., 1998) using ITS data. On the other hand, Sulaiman et al. (2003) reported a higher infection rate of 15.2% (13/85) in North American beavers trapped in streams and lowland areas of eastern Maryland (U.S.) were positive for these microsporidia, bearing six genotypes of *E. bieneusi* (denoted by the authors as WL7, WL8, WL9, WL12, WL13, WL15). Genotype D (denoted as WL8) was detected four times in beavers and its broad host specificity was confirmed by records in muskrat, raccoon and fox also in that study. Of the beaver genotypes recorded by Sulaiman and co-authors, WL7, WL8 and WL12 were found only in semi-aquatic animals (beaver, otter) and are regarded to be specific to these hosts given that no findings in other animals were reported since then (Li et al., 2019). Genotype D of *E. bieneusi* identified here in the beaver sample from the Topľa river (northeastern Slovakia) was before commonly recorded in various animals of the Slovak fauna - stray cats, wild boar, domestic pigeon and a captive chimpanzee in ZOO Bratislava (Sak et al., 2011; Němejc et al., 2014; Kváč et al., 2017; Holubová et al., 2024). It is one of the most frequently reported genotypes of *E. bieneusi* (Santín and Fayer, 2011; Li and Xiao, 2021) belonging to Group 1, regularly documented in domestic/wild animals, humans and water sources worldwide, suggesting a higher probability of its zoonotic and waterborne transmission (Li et al., 2019). Globally, in water sources, *E. bieneusi* was the most frequently isolated species in microsporidia (23 published reports), and genotype D was the most prevalent genetic variant (12 reports) (Rezaeian et al., 2023).

In another sample of fungal spores taken from the Velický brook (tributary of the Poprad River, north Slovakia), *E. intestinalis* was detected in this survey. No studies are currently available that have reported microsporidia of the genus *Encephalitozoon* in both Eurasian and North American beavers (Fayer et al., 2006; Wilczyńska et al., 2022). *E. intestinalis* is considered the second most prevalent *Encephalitozoon* species in humans and has been shown to occur in several mammalian species such as cattle, goat, donkey, pig, dog, cat, rabbit or mountain gorillas, coatis, red ruffed and ring-tailed lemurs (Sak and Kváč, 2021). To our knowledge, this is the first description of *E. intestinalis* in Eurasian beavers. In previous surveys carried out in Slovakia, *E. intestinalis* was detected in a low prevalence of 0.7% (1/280) in faecal samples from wild mice trapped in five sites of eastern Slovakia (Danišová et al., 2015). Unlike this, *E. intestinalis* spores were detected in the faeces of as much as 25 out of 27 sows (92.6%) that were clinically healthy and randomly selected from a herd in eastern Slovakia. This pointing to the pig as a potential source of human infection with microsporidia.

## 5 Conclusion

Conservation efforts to restore beaver populations should focus not only on host species, but also on their pathogens. The increased risk of zoonotic transmission of the pathogenic *E. bieneusi* (genotype D) and *E. intestinalis* microsporidia, and potentially zoonotic *C. proliferans* protozoa was observed in the collection site of the Topľa river. A previously unrecognized, new subtype of *Blastocystis* sp*.* was recorded in the collection site of the Danube river. A more detailed study of aquatic mammals and their parasites would further help to better understand the epidemiology and potential for zoonotic or cross-species transmission of the pathogens studied.

## CRediT authorship contribution statement

**Júlia Šmigová:** Writing – review & editing, Writing – original draft, Visualization, Methodology, Investigation, Formal analysis, Data curation, Conceptualization. **Viliam Šnábel:** Writing – review & editing, Visualization, Validation, Software, Methodology, Formal analysis, Data curation, Conceptualization. **Serena Cavallero:** Visualization, Software, Methodology. **Ľubomír Šmiga:** Writing – review & editing, Visualization, Formal analysis, Conceptualization. **Ingrid Papajová:** Resources, Project administration, Funding acquisition. **Bohumil Sak:** Writing – review & editing, Project administration, Methodology, Funding acquisition. **Nikola Holubová:** Methodology, Investigation. **Martin Kváč:** Writing – review & editing, Software, Resources, Project administration, Investigation, Funding acquisition.

## Consent to publish

Yes All authors have read and agreed to the published version of the manuscript.

## Data availability

The datasets in this study are available from the corresponding author upon reasonable request.

## Ethical standards

The study reported here was conducted in compliance with the relevant local laws and regulations.

## Funding

This work was supported by the 10.13039/501100005357Slovak Research and Development Agency under the contract no. APVV-18-0351 and VEGA 2/0069/25.

This research was funded by Grant Agency of the Czech Republic, grant numbers GACR 21-23773S and GACR 20-10706S.

## Declaration of interest statement

The authors declare that they have no known competing financial interests or personal relationships that could have appeared to influence the work reported in this paper.
